# Identification of Abundant and Functional dodecaRNAs (doRNAs) Derived from Ribosomal RNA

**DOI:** 10.3390/ijms22189757

**Published:** 2021-09-09

**Authors:** Marine Lambert, Abderrahim Benmoussa, Idrissa Diallo, Katheryn Ouellet-Boutin, Véronique Dorval, Nathalie Majeau, Charles Joly-Beauparlant, Arnaud Droit, Alain Bergeron, Bernard Têtu, Yves Fradet, Frédéric Pouliot, Patrick Provost

**Affiliations:** 1 CHU de Québec Research Center/CHUL Pavilion—Université Laval, 2705 boulevard Laurier, Quebec City, QC G1V 4G2, Canada; marine.lambert@mail.mcgill.ca (M.L.); abderrahim.benmoussa@umontreal.ca (A.B.); Idrissa.Diallo@crchudequebec.ulaval.ca (I.D.); katheryn.ouellet-boutin@crchudequebec.ulaval.ca (K.O.-B.); nathalie.majeau@crchudequebec.ulaval.ca (N.M.); Charles.Joly-Beauparlant@crchudequebec.ulaval.ca (C.J.-B.); arnaud.droit@crchudequebec.ulaval.ca (A.D.); Alain.Bergeron@crchudequebec.ulaval.ca (A.B.); bernard.tetu@fmed.ulaval.ca (B.T.); Yves.Fradet@crchudequebec.ulaval.ca (Y.F.); Frederic.pouliot@crchudequebec.ulaval.ca (F.P.); 2 Department of Microbiology, Infectious Diseases and Immunology, Université Laval, Quebec City, QC G1V 4G2, Canada; 3 Faculty of Medicine, Université Laval, Quebec City, QC G1V 0A6, Canada; 4 Department of Molecular Medicine, Université Laval, Quebec City, QC G1V 4G2, Canada; 5 Department of Surgery, Université Laval, Quebec City, QC G1R 2J6, Canada

**Keywords:** RNA sequencing, small RNA, non-coding RNA, RT-qPCR, 5.8S rRNA

## Abstract

Using a modified RNA-sequencing (RNA-seq) approach, we discovered a new family of unusually short RNAs mapping to ribosomal RNA 5.8S, which we named dodecaRNAs (doRNAs), according to the number of core nucleotides (12 nt) their members contain. Using a new quantitative detection method that we developed, we confirmed our RNA-seq data and determined that the minimal core doRNA sequence and its 13-nt variant C-doRNA (doRNA with a 5′ Cytosine) are the two most abundant doRNAs, which, together, may outnumber microRNAs. The C-doRNA/doRNA ratio is stable within species but differed between species. doRNA and C-doRNA are mainly cytoplasmic and interact with heterogeneous nuclear ribonucleoproteins (hnRNP) A0, A1 and A2B1, but not Argonaute 2. Reporter gene activity assays suggest that C-doRNA may function as a regulator of Annexin II receptor (AXIIR) expression. doRNAs are differentially expressed in prostate cancer cells/tissues and may control cell migration. These findings suggest that unusually short RNAs may be more abundant and important than previously thought.

## 1. Introduction

High-throughput sequencing (HTS) technologies revolutionized molecular biology and genetics by allowing the sequencing of entire genomes and transcriptomes [1,2,3,4,5,6]. The use of RNA sequencing (RNA-seq), in particular, markedly expanded the repertoire of non-coding RNA (ncRNA) species [7,8,9,10,11,12], which are now recognized as critical regulators of gene expression. ncRNAs have been reported, among other things, to control the binding of transcription factors and to regulate alternative splicing and messenger RNA (mRNA) translation [9,10,13,14,15], which allows cells to rapidly adjust their gene expression programming to respond and adapt to a changing environment, including cellular stress conditions [16]. Notably, ncRNAs are involved in cell proliferation and development, and thus, their impairment likely contributes to the etiology of various diseases, including cancer [17,18,19,20].

ncRNAs are classified according to their origin, length, and/or function [21,22,23,24]. Small RNAs (sRNAs) comprise transcripts less than 200 nucleotides (nt) in length. Small RNA sequences may be transcribed from dedicated sequences and promoters [25], or derived from several pre-existing RNA species, including mRNA introns or exons, transfer RNAs (tRNAs) or ribosomal RNAs (rRNAs) [26,27]. This yields an extremely diverse population of sRNAs most often involved in the specific recognition of nucleic acid targets through complementary base pairing [28,29].

Historically, the discovery of microRNAs (miRNAs, 19 to 24 nt) was delayed by half a century because of the dogma prevailing at the time stating that such short RNAs could not be biologically relevant, leading researchers to perceive them as mere degradation products [30,31]. Despite the now recognized importance of sRNAs and the lessons learned from the past, a comparable belief still exists today that arbitrarily draws the limit for function or interest to sRNAs longer than 16 nt. It is believed that any endogenous sequences shorter than 16 nt may not be specific, be mapped with confidence to the genome or have biological significance. Therefore, these are readily discarded either prior to library construction or from sequencing datasets in a systematic manner. This is done in order to improve the signal-to-noise ratio, improve the depth of sequencing or facilitate downstream computational analyses [32], allegedly without the risk of losing important information. These beliefs are strong and have tainted the standardized pipeline of most, if not all, HTS platforms and procedures currently available to researchers worldwide.

The very high abundance of rRNAs in biological samples—they may represent ~80% of total RNA of a cell—is also perceived with a negative a priori. Focusing on improving the sensitivity of HTS experiments, most researchers choose to eliminate rRNAs by using rRNA removal kits, which also eliminates the possibility of obtaining information on sRNAs derived from the most abundant cellular RNA. Known as small ribosomal RNAs (srRNAs), they form an emerging family of ncRNAs [27,33] that display essential functions in gene regulation and development [34,35]. Whether endogenous sRNA species shorter than 16 nt or derived from rRNA exist remains unknown.

Bino John’s laboratory had reported the characterization of viral and human RNAs that were unusually small (17 nt), shorter than canonical microRNAs [36]. Three years later, we reported the serendipitous discovery, in human platelets [37], of an RNA species half the length of an miRNA, which we termed semi-microRNA (smiRNA). The prototype of a 12-nt smiRNA was devoid of direct gene regulatory effects, but modulated the regulatory effects of the microRNA from which it was derived [37].

Together, these findings prompted us to expand our search for unusually short (<16 nt) RNA species by not excluding rRNA a priori and by analyzing the content of the RNA size window between 8 and 30 nt of 11 different biological samples from six different organisms by sRNA-seq (Lambert et al., manuscript submitted). Here, we report the discovery of a new family of functional dodecaRNAs (doRNAs) derived from ribosomal RNA, with a focus on the two major species: the minimal core 12-nt doRNA sequence and its +1-nt variant C-doRNA (doRNA with a 5′ Cytosine), and study their localisation, partners and function in human and mouse cells.

## 2. Results

### 2.1. sRNA-Seq Analyses Revealed the Existence of 12-nt and 13-nt sRNAs

In a previous study, we have used sRNA-seq analysis, and we investigated the sRNA profile (8–30 nt) of six different species (*H. sapiens*, *M. musculus*, *D. melanogaster*, *A. thaliana*, *S. pombe*, *S. cerevisiae*) from 11 samples, and revealed the existence of very small RNAs of discrete sizes, with a large part coming from rRNA (Lambert et al., manuscript submitted).

**Figure 1:**
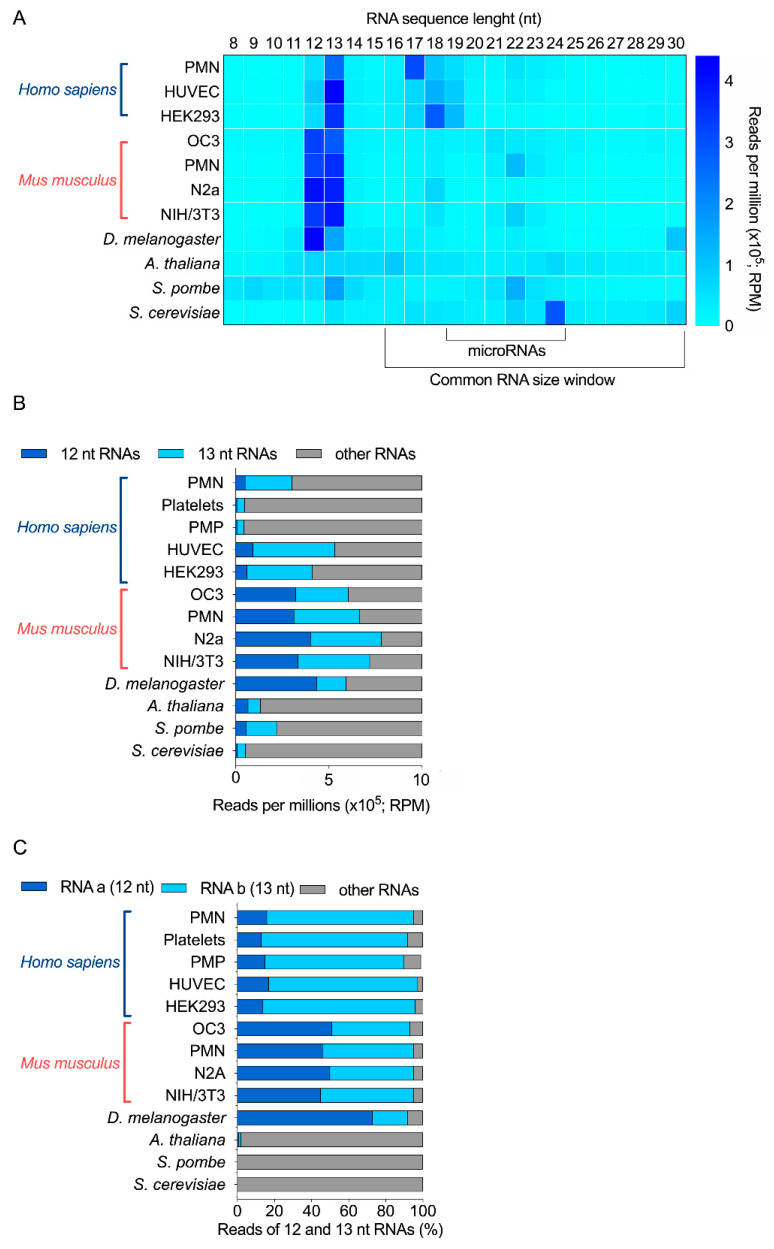
Relative abundance of 12 and 13 nt sRNA sequences obtained by sRNA-seq analyses of 11 different biological samples derived from 6 different species. (**A**) RPM abundance of RNA of 8 to 30 nt from 11 samples. (**B**) Relative abundance of 12-nt, 13-nt and other RNAs, expressed as RPM. (**C**) Relative proportion of the most abundant 12-nt RNA (RNA a) and 13-nt RNA (RNA b), compared with the other 12-nt and 13-nt RNAs detected by sRNA-seq (% of total reads). PMN, polymorphonuclear leukocytes; PMP, platelet-derived microparticles; HUVEC, human umbilical vein endothelial cells; HEK293, human embryonic kidney 293 cells; OC3, Old Cerebellum 3; N2a, mouse neuroblastoma cells; NIH/3T3, mouse embryonic fibroblast cells.

Together, in these RNA-seq data (Figure 1A), a relatively high abundance of 12-nt and 13-nt RNAs was observed in human, mouse, *Drosophila* and *S. pombe* samples, but not in *A. thaliana* and *S. cerevisiae*. Notably, 13-nt RNAs were more abundant than 12-nt RNAs in human and *S. pombe* samples, compared to mouse samples, where they seem to be equally represented. On the contrary, 12-nt RNAs were more abundant than 13-nt RNAs in *Drosophila* (Figure 1A,B). In fact, in these four organisms, these two-size ranges of RNAs (12 and 13 nt) represented between 22 to 74% of all RNAs sequenced in the 8 to 30 nt window of RNA length (Figure 1B); and the mouse neuronal N2a cell line is the most enriched in 12-nt and 13-nt RNAs, when added together.

Further bioinformatics analyses revealed that a unique 12-nt RNA sequence represented at least 70% of all 12-nt long RNAs for *H. sapiens*, *M. musculus* and *D. melanogaster*. We observed the same results for 13-nt RNAs (Supplementary Figure S1). This abundant 13-nt sequence was the same as the 12-nt RNA, but with an extra Cytosine (C) at its 5′ end. The sequence of the human and mouse 12-nt and 13-nt RNAs was identical. In total, two sequences accounted for 90% RNA reads (Figure 1C). On the opposite, while 12-nt and 13-nt RNAs are detected in *S. pombe*, no specific sequence was more abundant than the others.

Our sRNA-seq data support the existence of very small RNAs that are 12 nt or 13 nt long, more abundant than microRNAs, and with species and cell specificity. In particular, two main sequences represented most of these 12-nt and 13-nt RNAs in human, mouse and *Drosophila* samples, but were absent from *A. thaliana*, *S. pombe* and *S. cerevisiae*.

### 2.2. The Two Most Abundant 12-nt and 13-nt Sequences Likely Derive from 5.8S Ribosomal RNA

**Figure 2:**
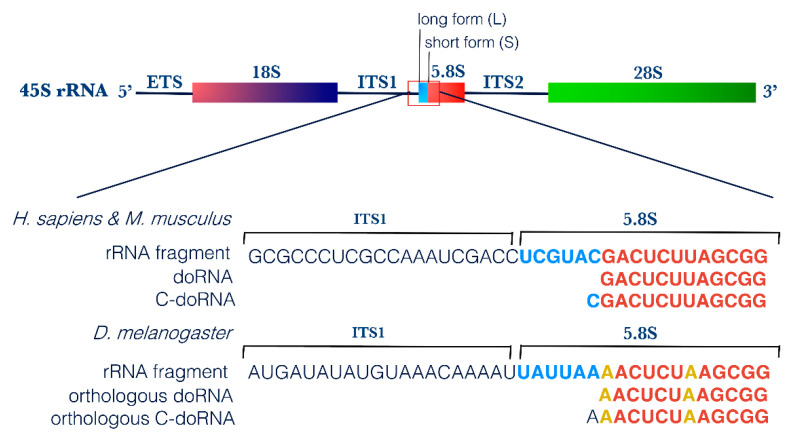
doRNA and C-doRNA sequences map to the 5′ end of the 5.8S rRNA. Schematic representation of doRNA and C-doRNA sequence alignment on the 45S rRNA in humans, mice and flies, using NCBI Nucleotide Reference Sequence (RefSeq) database. ETS, external transcribed spacer; ITS, internal transcribed spacer; rRNA, ribosomal RNA.

First, screening of these 12-nt and 13-nt RNA sequences for vector, adaptor, linker and primer contamination did not yield any positive match, excluding the possibility that they represent an artifact (on genomic and transcript database). Using NCBI BLAST, we mapped the two most abundant 12-nt and 13-nt RNA sequences to the transcriptome of each organism. In human, both sequences perfectly matched with ribosomal RNAs; the 5.8S rRNA and its longer 45S rRNA precursor. The same results were obtained when mapping these sequences to the murine transcriptome, as both are conserved between the two species. In *Drosophila*, despite the difference in nucleotide composition, the corresponding, equivalently abundant 12-nt and 13-nt RNA sequences also matched to *Drosophila*’s 5.8S and 45S rRNAs (Table 1). The orthologous 12-nt *Drosophila* RNA differed from the corresponding human and mouse sequences by 2 nt, whereas the 13-nt *Drosophila* RNA harbor an Adenine (A) at its 5′ end, instead of a Cytosine (C) for the human and mouse—in both cases, the extra 5′ nucleotide matched to the corresponding nucleotide on the longer 45S rRNA (Figure 2).

We propose to name this new RNA family dodecaRNAs (doRNAs), with respect to the number of core nucleotides (12 nt) their members contain. The most abundant 13-nt variant of doRNA harbors, in human and mouse, a C at its 5′ end and was consequently termed C-doRNA. We found that the doRNA sequence mapped directly to the 5′ end of the 5.8S rRNA (Figure 2). Thus, doRNAs might be formed through a specific and controlled cleavage of the 5.8S rRNA or transient rRNA precursors leading to 5.8S rRNA (e.g., 45S, 36S, 32S, 12S, 8S rRNAs). As rRNAs are the most abundant RNAs in cells (80% of all RNAs) [38], it is very likely that the doRNA and C-doRNA sequences, which are similarly overly abundant (e.g., compared to microRNAs) in the 8- to 30-nt window of RNA sizes, are sRNA fragments derived from rRNA (rRFs) [27].

This possibility is reinforced by the presence, in human and mouse 5.8S rRNA sequences, of a recurrent 2′-O-ribose methylation of the Uracil (U) positioned immediately downstream to the 3′ nucleotide of doRNAs [39]. This feature suggests that the modified U may be a signal or a determinant for the generation of their 3′ extremity (Figure 2). This feature reinforces the possibility that doRNAs originate from the 5.8S rRNA or longer precursors containing it.

### 2.3. The C-doRNA/doRNA Ratio Differs between Species

**Figure 3:**
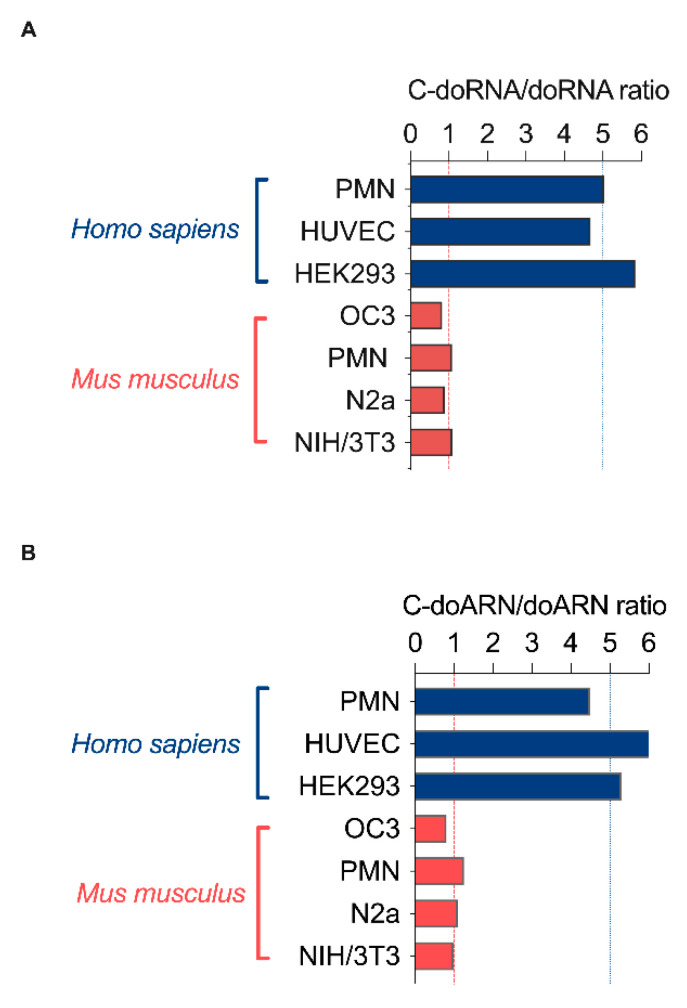
C-doRNA/doRNA ratio in human and mouse samples. (**A**) Calculated C-doRNA/doRNA ratio in human and mouse samples from the read count in RNA sequencing. (**B**) Calculated C-doRNA/doRNA ratio in human and mouse samples analyzed by splinted ligation RT-qPCR. Copy number of each RNA was calculated using a standard curve produced by a serial dilution of the synthetic form of each RNA. PMN, polymorphonuclear leukocytes; HUVEC, human umbilical vein endothelial cells; HEK293, human embryonic kidney 293 cells; OC3, old Cerebellum 3; N2a, mouse neuroblastoma cells; NIH/3T3, mouse embryonic fibroblast cells.

During our analyses, we observed a trend in the relative abundance of C-doRNA versus doRNA, which prompted us to calculate the C-doRNA/doRNA ratio in each sample. We found that this ratio was similar between samples derived from the same species but differed from one species to another. The calculated C-doRNA/doRNA ratio was ~5 in human samples, compared to ~1 in mouse samples and ~0.25 in the *Drosophila* sample (Figure 3A and Figure S2). The difference between species may highlight dissimilarities in their biogenesis in terms of enzyme(s)/cofactor(s) involved, precursor(s), processing, stability, interacting proteins and function. As this ratio increases from flies to mice to humans, it may also hint at an evolutionary promotion of C-doRNA expression over that of doRNA and at a potential implication of the 5′ C in C-doRNA function. The increasing C-doRNA/doRNA ratio may also be related to the increasing complexity of living organisms, especially bilaterians.

We confirmed that these two abundant, unusually short RNAs were not an artefact of the experiment (e.g., library construction) and validated their existence by using a new RT-qPCR method aimed to specifically quantitate doRNA and C-doRNA levels, as described in Lambert et al. (manuscript submitted). This method was used to confirm doRNA and C-doRNA expression in various human and mouse samples (Lambert et al., manuscript submitted) as well as their overly abundance compared to two microRNAs. Here, we started to document the C-doRNA/doRNA differential enrichment between species thanks to the copy number obtained by RT-qPCR. This later was still comparable between the cells of the same species, but differed between species, as the RNA-seq results (a C-doRNA/doRNA ratio of ~1 in mice and of ~5 in humans, as shown in Figure 3B). These findings were confirmed by RT-qPCR analysis of samples from different cultures of human HEK293 and mouse N2a cells, in which, again, the C-doRNA/doRNA ratio was statistically different and around 5 and 1 respectively (Supplementary Figure S3).

### 2.4. doRNA and C-doRNA Interact with Three Proteins from the hnRNP Family

**Figure 4:**
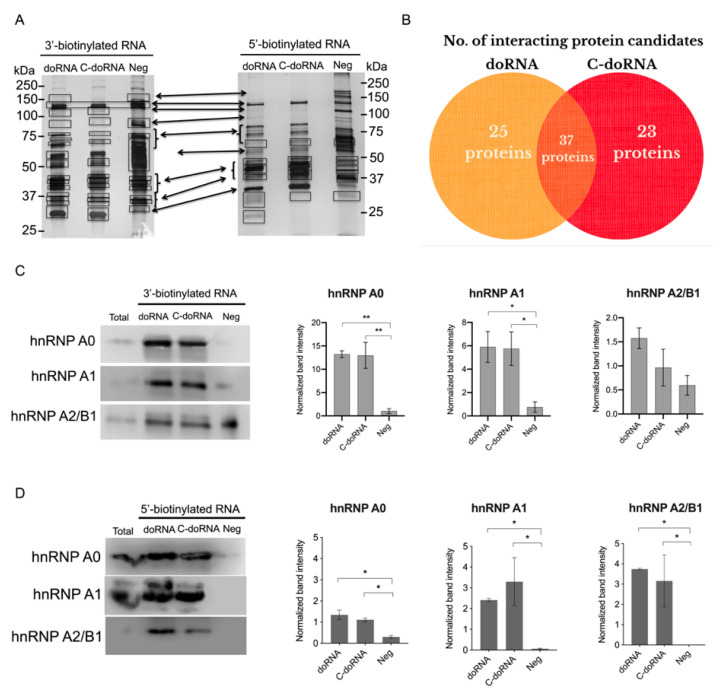
Identification of hnRNP A0, A1 and A2B1 as doRNA and C-doRNA-interacting proteins. (**A**) Pull-down experiments using 5′ or 3′ biotinylated doRNA, C-doRNA or negative RNA (Neg) and mouse brain extracts, using streptavidin beads. (**B**) Venn diagram showing the number of proteins interacting with doRNA and/or C-doRNA, and not with Neg RNA. (**C**,**D**) The most promising interacting protein candidates identified by LC/MS-MS were validated by Western blot analysis of the pull-downs using monoclonal antibodies against hnRNP A0, A1 and A2B1. Band intensity was quantitated using ImageJ (*n* = 3 independent experiments). * *p* < 0.05, ** *p* = 0.0044 (one-way ANOVA with Holm Sidak’s post-hoc test).

To identify potential protein partners of doRNA and C-doRNA, we used a pull-down assay based on 3′ or 5′ biotinylated sRNAs (doRNA, C-doRNA and a negative RNA) [40] and streptavidin beads, followed by protein identification by LC/MS-MS (Figure 4A). Considering protein candidates with more than 3 peptide matches and absent from the negative RNA sample, we found that 25 proteins were associated specifically with doRNA, 23 specifically with C-doRNA, from a total of 85 proteins associated with either doRNA or C-doRNA (Figure 4B). Among these proteins, three members of the heterogeneous nuclear ribonucleoproteins (hnRNP) family were particularly abundant: hnRNP A0, A1 and A2/B1, corresponding to the ∼30, 32 and 37-kDa bands, respectively. For each of these proteins, a minimum of 28 peptides, covering at least 64% of the amino acid sequence, were obtained (Figure 4A,B).

We validated the interaction between the doRNA, C-doRNA and these three hnRNPs by using the same pull-down strategy, followed by Western blotting. Notably, hnRNP A0 was more enriched in the pulled-down doRNA and C-doRNA when these sRNAs were biotinylated at their 3′ rather than their 5′ end (Figure 4C,D). The major reduction of hnRNP A0 protein association when the biotin moiety was transferred from the 3′ to the 5′ end suggests that hnRNP A0 preferentially binds doRNA and C-doRNA through the 5′ end.

**Figure 5:**
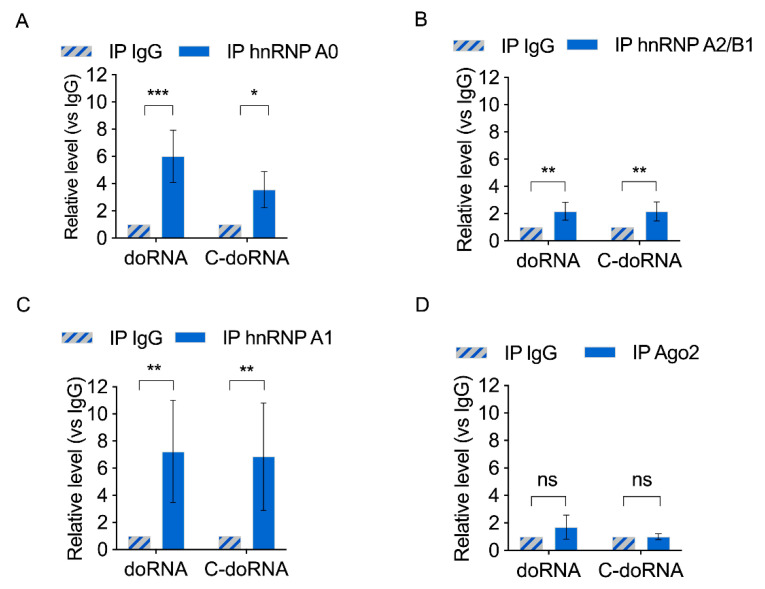
Detection of doRNA and C-doRNA in hnRNP A0, A1 and A2B1, but not Ago2, complexes by reciprocal RNA immunoprecipitation. (**A**–**D**) Immunoprecipitation of hnRNP A0 (**A**), A1 (**B**) or A2B1 (**C**) proteins, or Ago2 (**D**) proteins, from mouse brain extracts, followed by splinted ligation RT-qPCR detection of doRNA and C-doRNA. Changes in the level of coimmunoprecipitating doRNA and C-doRNA was expressed as fold change versus the control IgG IP (*n* = 3 independent experiments). * *p* < 0.05; ** *p* < 0.005; *** *p* < 0.0005 (Student’s *t*-test).

We next performed the reciprocal experiment, in which endogenous hnRNP A0, A1 and A2/B1 were immunoprecipitated, and doRNA and C-doRNA were quantitated by RT-qPCR. Both doRNA and C-doRNA were significantly enriched in the hnRNP A0, A1 and A2B1 protein immunoprecipitates, compared to the normal IgG control immunoprecipitate (Figure 5A–C). Neither doRNA nor C-doRNA could be detected in immunoprecipitates of the miRNA effector protein Ago2 (Figure 5D). Quantitation of miR-25 and miR-30a in these immunoprecipitates confirmed their association with Ago2, but not with hnRNP A0, A1 and A2/B1 (Supplementary Figure S4). The identification of hnRNP A0, A1 and A2/B1 proteins as doRNA and C-doRNA-interacting proteins in mouse brain cortex suggest that doRNA and C-doRNA may function as hnRNP complexes.

### 2.5. The doRNA and C-doRNA Were Broadly Localized at the Cytoplasm

**Figure 6:**
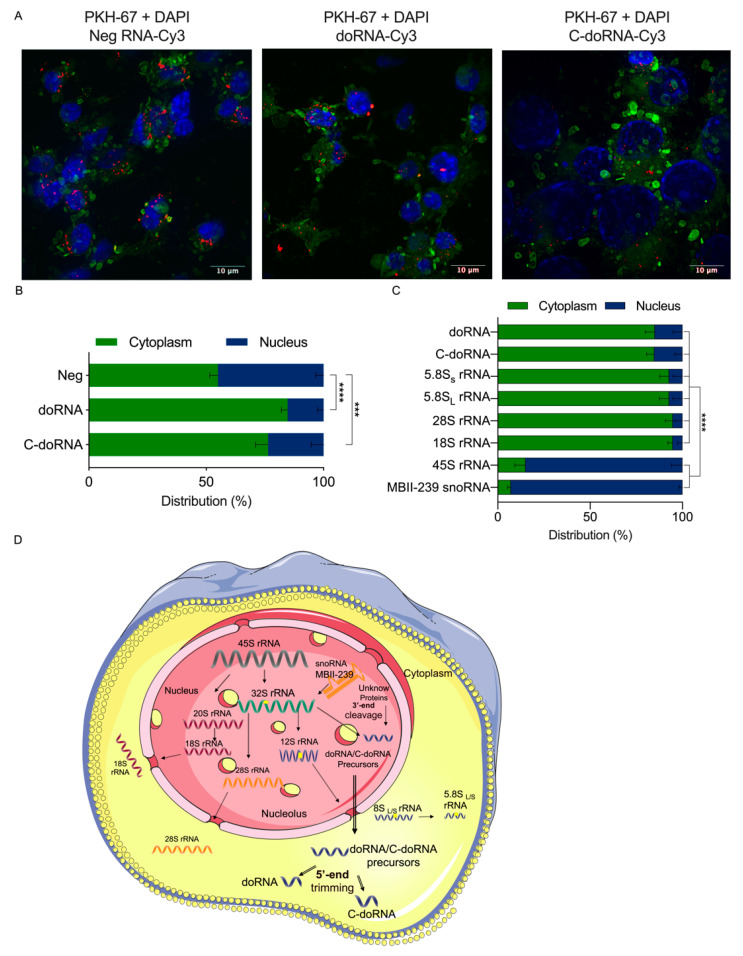
Cytoplasmic/perinuclear localization of fluorescently labeled doRNA and C-doRNA after transfection in cultured neuronal N2a cells. (**A**) Localization of doRNA, C-doRNA or negative RNA (Neg; control) coupled at their 3′ end with the Cy3 fluorophore (in red) in transfected N2a cells 24 h prior to confocal fluorescence microscopy. Cell membranes were labeled with the marker PKH-67 (in green), whereas cell nuclei were labeled with 4′,6-diamidino-2-phenylindole (DAPI). RNA localization was visualized using a confocal microscope with a 63× *g* magnification. (**B**) Quantitation of the red dots corresponding to doRNA, C-doRNA or negative RNA in the nuclear or cytoplasmic compartments (*n* = 30 cells, from 3 independent experiments). *** *p* = 0.0006, **** *p* < 0.0001 (two-way ANOVA with Holm Sidak’s post-hoc test). (**C**) RT-qPCR quantitation of doRNA, C-doRNA, rRNAs 28S, 5.8Sl, 5.8Sl, 18S, their 45S rRNA precursor, and the MBII-239 snoRNA control in the cytoplasmic and nuclear fractions of N2a cells (*n* = 4 independent experiments). **** *p* < 0.0001 (two-way ANOVA with Holm Sidak’s post-hoc test). (**D**) Schematic representation of the proposed cellular localization of doRNA and C-doRNA relative to their precursors in mammalian cells.

If C-doRNA and doRNA were to be functional, especially by their interaction with the proteins hnRNP A0, A1 and A2B1, which are able to shuttle between nucleus and cytoplasm, their location within cells would be crucial to their function. To investigate this avenue, we used synthetic doRNA, C-doRNA and negative control RNA coupled with a Cy3 fluorophore and monitored their intracellular localization 24 h after transfection in cultured N2a cells. doRNA and C-doRNA were localized mainly to the cytoplasm, near the nucleus, and often visualized as punctate staining (Figure 6A). In opposition, the negative control RNA was more evenly and homogeneously distributed between and within the compartments. Quantitative assessment showed doRNA and C-doRNA to be preferentially located in the cytoplasm of cells (Figure 6B).

To confirm and transpose these results to endogenous doRNA and C-doRNA, we performed subcellular fractionation of cultured N2a cells followed by Western blot analyses. PARP-1 and GAPDH were used as nuclear and cytoplasmic protein markers, respectively, to confirm the quality of the fractionation (Supplementary Figure S5). The longer rRNA precursors of doRNA and C-doRNA, namely 28S, 18S, 5.8S_S_ and 5.8Sl rRNAs, were mainly localized to the cytoplasm. Expectedly, 45S rRNA was localized to the nucleus. We also localized mouse small nucleolar RNA (snoRNA) MBII-239, which is responsible for the 2′-O ribose methylation of the U-14 in the 5.8S rRNA [41], almost exclusively to the nucleus. Both endogenous doRNA and C-doRNA were localized mainly, but not exclusively, to the cytoplasmic compartment (Figure 6C), corroborating the cytoplasmic localization of the Cy3 derivatives shown in Figure 6A,B.

The nuclear enrichment of the rRNA precursors and the cytoplasmic enrichment of both doRNA and C-doRNA are consistent with the rRNA precursors being processed in the nucleus and with both doRNA and C-doRNA products being exported to the cytoplasm, probably as ribonucleoprotein complexes.

### 2.6. C-doRNA Impairs Reporter Gene Expression Controlled by the AXIIR 5′ UTR

**Figure 7:**
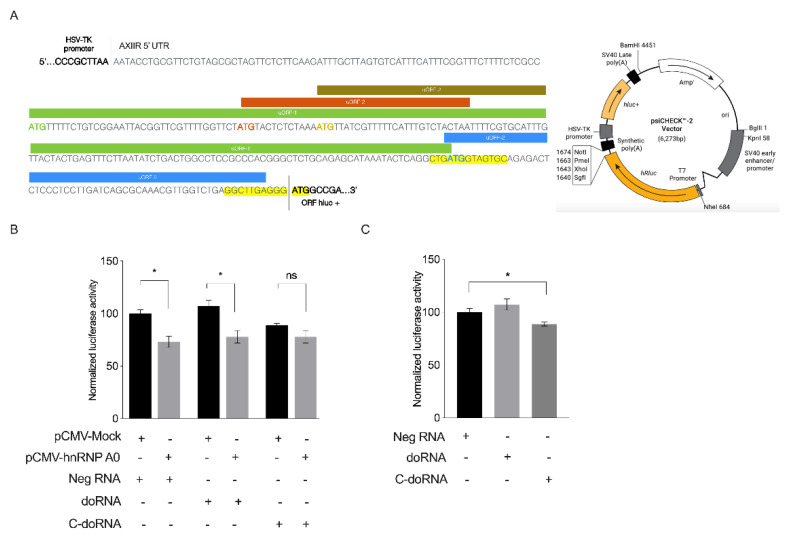
C-doRNA slightly impairs reporter gene expression controlled by the Annexin II receptor (AXIIR) 5′ UTR. (**A**) Schematic representation of AXIIR 5′ UTR sequence cloned into the dual-luciferase reporter gene expression psiCHECK-2 vector. (**B**) Synthetic doRNA, C-doRNA or negative RNA (Neg) control was transfected in cultured N2a cells overexpressing (or not; empty vector) hnRNP A0 as well as the Firefly (Fluc; hluc+) and Renilla (Rluc; hRluc, control) luciferase reporter genes. Fluc activity was normalized on Rluc activity, and the data expressed as % of control (Neg RNA + pCMV-Mock); *n* = 3 independent experiments. * *p* < 0.05; ns, not significant (two-way ANOVA with Holm Sidak’s post-hoc test). (**C**) doRNA, C-doRNA or negative RNA (Neg) control was transfected in cultured N2a cells with the psiCHECK-2 vector. Fluc activity was normalized on Rluc activity, and the data expressed as % of the Neg control; *n* = 3 independent experiments. * *p* < 0.05; ns, not significant (two-way ANOVA with Holm Sidak’s post-hoc test).

hnRNP A0 protein was previously reported to regulate the expression of Annexin II receptor (AXIIR) via upstream open reading frames (uORFs) present in the mRNA 5′ UTR of this receptor [42]. This led us to posit whether doRNA or C-doRNA could be involved in the regulation of AXIIR expression. To explore that possibility, we used a dual-luciferase gene assay using a reporter construct in which the 5′ UTR sequence of AXIIR was inserted upstream of the Fluc ORF, with Rluc as a normalization control (Figure 7A). The vector psiCHECK-2-AXIIR 5′ UTR was co-transfected with the vector pCMV-hnRNP A0 or its negative pCMV-Mock control in cultured mouse N2a cells 24 h before transfecting the cells with doRNA, C-doRNA or negative control RNA. Fluc and Rluc activity was measured 24 h later.

As expected, overexpression of hnRNP A0 protein significantly decreased reporter gene expression placed under the control of the AXIIR 5′ UTR (Figure 7B). This inhibition persisted upon addition of doRNA or C-doRNA. Transfection of C-doRNA alone impaired reporter gene expression regulated by AXIIR 5′UTR, an effect that was also observed in transiently transfected N2a cells expressing endogenous levels of hnRNP A0 protein (Figure 7C). These results suggest that doRNA and C-doRNA exert distinct gene regulatory functions, with C-doRNA possibly regulating AXIIR expression.

### 2.7. doRNA Levels, but Not C-doRNA, Are Decreased in Prostate Cancer Samples and Cell Lines

AXIIR protein is a membrane receptor sensitive to androgens and is particularly involved in prostate cancer [43,44]. Therefore, we decided to document the level of doRNA and C-doRNA expression in normal (RWPE-1) and cancerous (LNCaP, VCaP, PC3, DU145, 22Rv1, LaPC4) prostate cell lines using splinted ligation RT-qPCR. We observed a trend towards lower doRNA and C-doRNA levels in two hormone-sensitive, LNCaP and VCaP, prostate cancer cells, compared to the normal RWPE-1 cells. The trends reached statistical significance in three cases out of four (Supplementary Figure S6), probably due to the small number of LNCaP samples *(n* = 3) analyzed.

**Figure 8:**
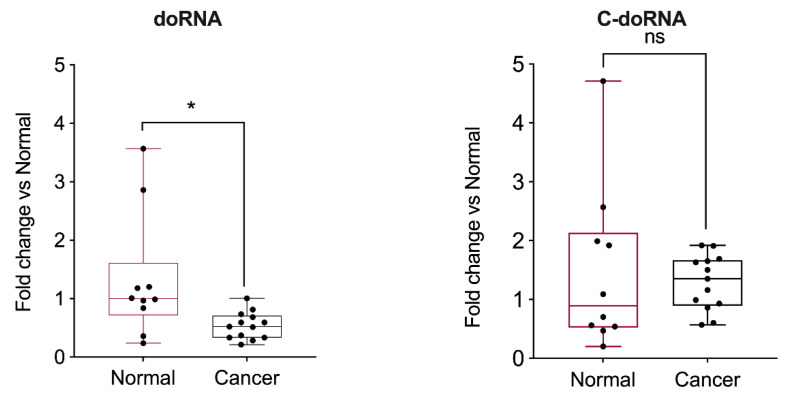
C-doRNA levels do not varied whereas doRNA levels are reduced in prostate cancer tissues. doRNA and C-doRNA were quantitated in normal (*n* = 10) and cancerous (*n* = 13) prostate tissue samples. * *p* < 0.005; ns, not significant (Student’s *t*-test).

These results were transposed to human normal and cancerous prostate tissue samples, in which we detected significantly lower doRNA levels in prostate cancer tissues compared to normal prostate tissues (Figure 8). However, no difference in C-doRNA levels was observed. Although doRNA may not regulate AXIIR expression via its 5′ UTR, it may still be involved in prostate cancer in other ways.

### 2.8. C-doRNA Reduces Cellular Migration/Proliferation of Cancer and Normal Prostate Cells

Considering that doRNA levels vary in prostate cancer tissues and that C-doRNA may regulate expression of AXIIR, which is involved in prostate cancer [44], we asked whether doRNA or C-doRNA could modulate cell migration and/or proliferation, major drivers of cancer. To explore this scenario, we performed scratch assays using transiently transfected RWPE-1 normal prostate and LNCaP prostate cancer cells.

**Figure 9:**
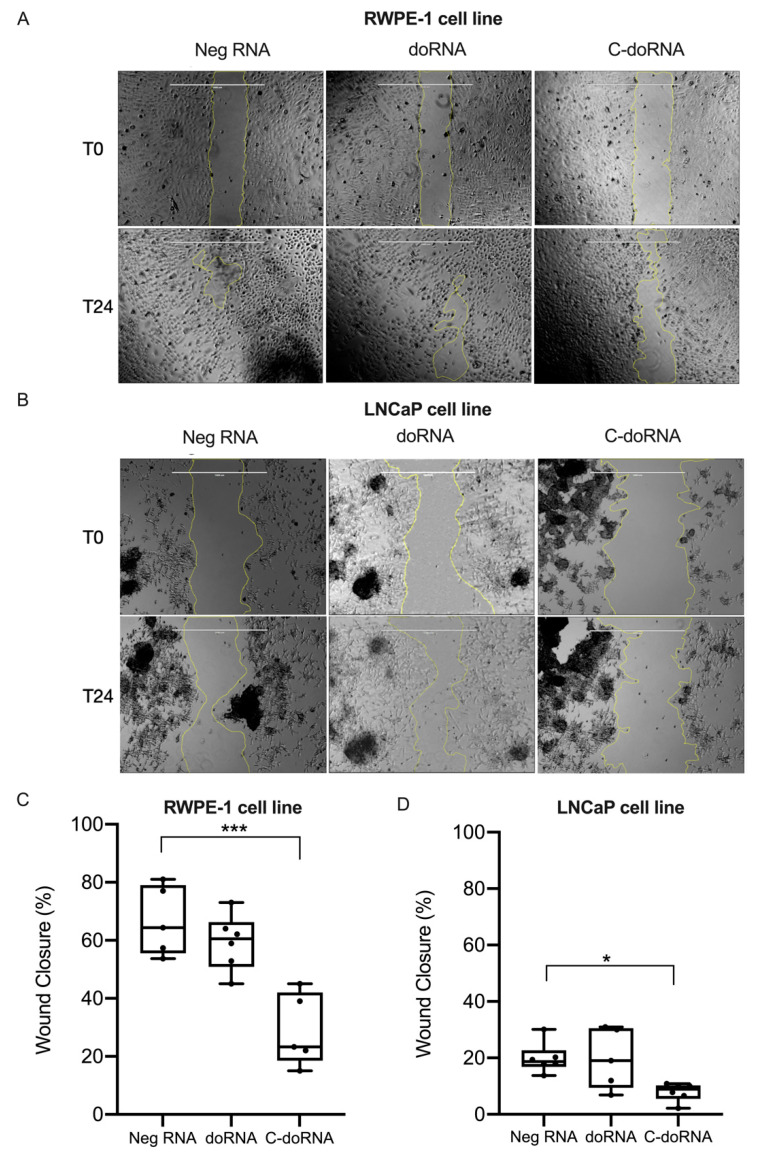
C-doRNA reduces wound closure upon scratching of confluent culture of prostate cells. (**A**–**D**) RWPE-1 (**A**,**C**) and LNCaP (**B**,**D**) cells were transfected with negative RNA (Neg), doRNA, C-doRNA or sponge RNA. (**A**,**B**) Images were captured by a camera coupled to a view INV100 microscope, with 40× magnification, immediately after (T0) and 24 h after (T24) performing a scratch. Scale bar are 1000µm for all pictures. (**C**,**D**) The images were analyzed using the ImageJ software to evaluate the closure of the scratch by quantitating the areas devoid of cell coverage. *n* = 6 independent experiments. * *p* < 0.05; *** *p* < 0.0005 (two-way ANOVA with Holm Sidak’s post-hoc test).

doRNA transfection had no effect on the migration/proliferation of RWPE-1 (Figure 9A,C) or LNCaP (Figure 9B,D) cells, compared to negative RNA control transfection. However, C-doRNA transfection induced a decrease in cell migration/proliferation in both normal and cancerous prostate cell lines (Figure 9). These results suggest that C-doRNA may be involved in prostate cell migration/proliferation under either normal or pathological conditions.

## 3. Discussion

The two major forms of the new family of doRNAs, the minimal core 12-nt doRNA sequence and its +1 nt variant harboring a 5′ C (C-doRNA), map perfectly to the 5.8S rRNA sequence, from which they most likely derive. The 5′ end of C-doRNA is identical to that of the mature 5.8S rRNA sequence, suggesting that both doRNAs may be produced by the same ribonuclease or may derive from the mature form of 5.8S rRNA. The conservation of doRNA and C-doRNA sequence length, relative abundance and perfect mapping to the 5.8S rRNA, despite a 2 to 3-nt difference between the human or mouse and *Drosophila* sequences, further supports their rRNA origin. When considering also the very small RNA profile of *Arabidopsis*, fission and budding yeasts (from which doRNAs appear to be absent), one can only speculate at this time whether doRNAs appeared during the course of evolution along bilaterians or were involved in their emergence.

Some variants of doRNAs longer than 12 nt harbor nucleotide substitutions (Supplementary Table S1, colored nucleotides), suggesting that they may originate from different regions of the genome. This nucleotide variability may derive from the corresponding diversity in rDNA sequences [45,46,47,48]. Indeed, human and mouse genomes encode hundreds of rDNA operon copies, which are arranged in arrays on five chromosomes (13, 14, 15, 21, and 22 in human; 12, 15, 17, 18, and 19 in mouse) [48,49,50]. This high number of repetitions might thus explain the heterogeneity and relative abundance of the sequence variants of doRNAs, as well as the abundance of doRNAs themselves.

In human, mouse and *Drosophila*, doRNA-containing sequences longer than 12 nt are solely single nt extensions of their 5′ end encompassing the 3′ end of the internal transcribed spacer 1 (ITS1) when reaching 18 nt (Supplementary Table S1; also refer to Figure 2). No doRNA-containing sequences extended at their 3′ end were found (Supplementary Table S1), meaning that all doRNAs bear a G at their 3′ end. This feature strongly argues for an enzyme-mediated, specific cleavage of a 5.8S rRNA-containing precursor at the phosphodiester bond between G13 and U14.

First suspected are endoribonuclease enzymes already known to be involved in 5.8S rRNA maturation. In eukaryotes, 5.8S rRNA maturation proceeds via two pathways yielding two 5.8S rRNAs differing by their 5′ end: a short form of 5.8S (5.8S_S_) and a long form of 5.8S (5.8Sl) [51,52]. The most abundant 5.8S_S_ rRNA is generated through an endonucleolytic cleavage of the 27S pre-rRNA by ribonuclease MRP, followed by an exonucleolytic trimming by exoribonucleases Rat1p and Rrp17p.13 [52,53,54,55,56]. Alternatively, the less abundant 5.8S_L_ rRNA is produced by direct cleavage of the 27S rRNA precursor [57]. In human and mouse cells, XRN2 (yeast Rat1p) is required for tailoring the 5′ end of 32S rRNA [52,57], suggesting that such a 5′→3′ exoribonuclease might process longer 5.8S rRNA precursors into doRNAs of various length.

Endoribonucleases are often assisted by cofactors or guided by structural elements, sRNAs or chemical modifications to achieve cleavage at specific sites [58,59]. Although decades of investigation have identified most of the rRNA-modified nucleotides [60], outlined their chemical and structural properties [61] and unveiled their function in rRNA maturation [62], the detailed functional and regulatory consequences of these mechanisms remain unknown. It is known that rRNA precursors are extensively modified during the maturation process, mostly by methylation of the 2′ hydroxyl group of specific ribose and conversion of specific uridine residues to pseudouridine [63] (Supplementary Figure S7). It is revealing that nt 14 in 5.8S rRNA, which is next to the 3’ end nt (G13) of doRNAs is a Uracil, which is subjected to 2′-O-methylation [41,64,65] (Supplementary Figures S7 and S8). It is tempting to hypothesize that the 2′-O-methylation of U14, a modification present in mature 5.8S rRNA [41,66], may be a major determinant of the 3′ end of doRNAs. Interestingly, murine snoRNA MBII-239 (HBII-239 in human) [65] has been described and predicted to guide 2′-O-ribose methylation of 5.8S rRNA on residue U14 [65]. The possibility that cleavage into doRNAs, of mature 5.8S rRNA or that of precursor(s) containing it, is modulated by snoRNA-mediated 2′-O-methylation of U14 [65], which would either prevent it or allow it through guidance of an endoribonuclease to its specific activity site [67], is attractive.

A link between the suspected snoRNA and cancer was recently established, as human HBII-239 was positively associated with a good prognostic in angio-immunoblastic T-cell lymphoma (AITL) and peripherical T-cell lymphoma-not otherwise specified (PTCL-NOS) [68]. Indeed, snoRNAs appear to be dysregulated in cancer [69], showing a global increase in breast and prostate cancer [70]. The reduced levels of doRNA detected in prostate cancer cells and tissues in the present study suggest that the relationship between snoRNA-induced 5.8S rRNA U14 2′-O-methylation and generation of doRNAs, if there is one, may be inversely correlated.

In contrast to their invariable 3′ end, the 5′ end of doRNAs is highly variable, and may be generated by an exonuclease—a family of enzymes known to digest their substrates one nucleotide at a time [71] as hinted by the stair-shaped alignment of doRNA-containing reads on their 5′ side (Supplementary Table S1). Whether differences in the identity of the 5′ nt translates into differences in protein partners or function remains to be determined. This seems to be the case, as evidenced by the 25 partner proteins for doRNA only and the 23 for C-doRNA only, identified in the proteomics analysis (Supplementary Table S2). The order in which the 5′ and 3′ ends of doRNAs are generated and the enzymes that may be involved in the process also remain unknown.

The 5.8S and 28S rRNAs hybridize through three regions within the ribonucleoprotein complex-forming ribosomes [66], and one of those base-paired regions involves the doRNA-containing sequence of 5.8S rRNA (Supplementary Figure S8). Therefore, the mature doRNA and C-doRNA sequences may bind to the 28S rRNA, disrupt the 5.8S-28S rRNA interaction and affect the structural conformation and function of the ribosome [66]. 5.8S rRNA has a role in the translocation of the ribosome during translation [72]. Together, these findings suggest that doRNA and C-doRNA may compete for the 28S rRNA binding site, compromise 5.8S rRNA function and interfere with ribosome translocation, mRNA translation and protein synthesis, which may provide the molecular basis of their role as global regulators (i.e., suppressors) of cellular protein synthesis.

Among those elements are the three hnRNP A0, A1 and A2/B1 interacting with doRNAs and implicated in translation regulation and mRNA stability, among other activities [42,73]. Zhang et al. [42] demonstrated that hnRNP A0 and A2/B1 could jointly regulate AXIIR mRNA translation. In this context, we have shown that C-doRNA slightly impaired gene expression controlled by the AXIIR 5′ UTR, supporting its ability to regulate specific mRNAs. Therefore, it is tempting to speculate that C-doRNA and/or doRNA may act through modulation of hnRNP function, either as an hnRNP complex or by competing with their mRNA binding, or a combination thereof. Especially, in the pull-down experiment, we saw a major reduction of hnRNP A0 protein association when the biotin moiety was transferred from the 3′ to the 5′ end, suggesting that hnRNP A0 preferentially binds doRNA and C-doRNA through the 5′ end. Based on these results, each of the doRNA partner proteins identified in LC/MS-MS could provide a better understanding of the function of these RNAs. In particular, our LC/MS-MS data suggest that some proteins were able to specifically bind doRNA and not C-doRNA; as well, C-doRNA bound protein partners that were not found in doRNA pull-down. Further studies may help differentiate the gene regulatory effects of doRNAs.

As described by Blenkiron et al. [74], the human Y-box binding protein 1 (YB-1) could bind SNORD71, and also associate with hnRNP A1. Furthermore, YB-1 has been reported to be associated with ncRNAs and to alter proliferation of prostate cancer cells [75]. Considering our results revealing a role for C-doRNA in prostate cell migration/proliferation and its interaction with hnRNA proteins A0, A1 and A2/B1, it would be interesting to better understand the dynamics of interaction between these RNAs (C-doRNAs, SNORD71) and proteins (YB-1, hnNRP A1 and A/B), and the role that their ribonucleoprotein complexes might play in cellular homeostasis and prostate cancer pathogenesis.

Gene ontology (GO) analysis of the 80+ doRNA and/or C-doRNA protein partner candidates identified by proteomics analyses suggests their involvement in RNA splicing, mRNA stability, translation regulation and stress granule biology (Supplementary Table S2). doRNAs may contribute to these processes as members of RNP complexes. We observed a significant enrichment of doRNA-interacting proteins in “protein localization to adherent junctions” (Supplementary Table S2), which, added to our bioinformatics analyses of binding sites for doRNAs in mRNA 5′ UTRs (Supplementary Table S3), may help elucidate C-doRNA function in cell migration.

Further investigations are required to better understand the role, importance, function and mechanisms underlying the cell and molecular biology of doRNAs. To this end, knock-down or neutralization usingand RNAi-type approaches might be very informative. However, targeting of these unusually short and abundant doRNA sequences, using an adapted sponging strategy similar to that used to inactivate microRNAs [76], poses a significant challenge in terms of specificity and efficacy, especially considering that their 5.8S rRNA precursors contain the very same sequence—it may thus be difficult to distinguish doRNA inactivation from that of 5.8S or 45S rRNA. As for CRISPR/Cas9, it may not be a suitable approach to inactivate the 200 copies of the rRNA-encoding rrn gene present in the human genome. Such studies are likely to provide new insights and perspectives on their possible use as biomarkers or therapeutic targets or agents, and on whether they represent the missing component in some yet to untangle cellular and molecular processes in health and disease conditions.

## 4. Materials and Methods

### 4.1. Ethical Statement

#### 4.1.1. Human Blood Samples 

Collection of venous blood from healthy volunteers (adult Caucasians of both sexes from the immediate region of Quebec City) was approved by our CHU de Quebec-Université Laval Research human ethics committee (Approval Code: 2015-2103, B14-08-2103; Approval Date: 17 October 2017). The participants provided their written informed consent to participate in this study, in accordance with the Declaration of Helsinki.

#### 4.1.2. Mouse Tissue Samples

This study was carried out in accordance with the guidelines, regulations and requirements of the Canadian Council of Animal Care for Animals Used for Scientific Purposes. All experiments were performed in accordance with the latest guidelines and using a protocol approved by the Université Laval Animal Welfare Committee (Approval Code: 2014056-4; Approval Date: 11 May 2017). Well-being of the animals was monitored twice a day by trained animal facility services, and any signs of distress or pain were immediately reported to the veterinary services and treated, as per the ethical and veterinary guidelines.

#### 4.1.3. Human Prostate Tissue Samples 

RNA samples of normal prostate and prostate tumors used in this study were obtained from the URO-1 biobank of the CHU de Québec-Université Laval. This study was approved by the CHU de Quebec-Université Laval Research Ethics Committee (Approval Code: 2017-3503; Approval Date: 11 February 2017) and performed in accordance with the Declaration of Helsinki.

### 4.2. Biological Samples

#### 4.2.1. Primary Human Cells 

Human blood polymorphonuclear (PMN) leukocytes, platelets and platelet-derived microparticles (MPs) were isolated from venous blood collected from four healthy donors and pooled, as described in Laffont et al. [76].

#### 4.2.2. Cultured Human Cells 

Human umbilical vein endothelial cells (HUVEC; Stem Cell Technologies, Vancouver, BC, Canada) were cultured in endothelial growth medium (Lonza, Basel, Switzerland) supplemented with bovine brain extract (Lonza, Basel, Switzerland) and maintained at 37 °C under 5% CO_2_. For all experiments, HUVEC were used between passages 2 to 6. Cultured human embryonic kidney 293 (HEK293; ATCC, Manassas, VA, USA) were maintained in Dulbecco’s modified Eagle’s medium (DMEM) supplemented with 10% (*v*/*v*) fetal bovine serum (FBS), 1 mM sodium pyruvate, 100 units/mL penicillin, 100 μg/mL streptomycin, and 2 mM L-glutamine in a humidified incubator under 5% CO_2_ at 37 °C. The normal prostate (RWPE-1) and prostate cancer (VCaP, LaPC4, 22Rv1, DU145, PC-3 and LNCaP) cell lines were cultured in their appropriate medium (Supplementary Materials and Methods) and supplemented with 10% (*v*/*v*) fetal bovine serum (FBS, System BioScience^®^, Palo Alto, CA, USA), 1 mM sodium pyruvate, 100 units/mL penicillin, 100 μg/mL streptomycin, and 2 mM L-glutamine in a humidified incubator under 5% CO_2_ at 37 °C.

#### 4.2.3. Human Prostate Tissue Samples 

Total RNA from 10 normal and 13 cancerous prostate tissues were extracted using either the Absolutely RNA miRNA kit (Agilent Technologies, Waldbronn, Germany) or the mirVana miRNA isolation kit (Ambion Inc., Austin, TX, USA). The RNA samples were provided by the URO-1 biobank.

#### 4.2.4. Primary Mouse Cells and Tissues 

Mouse blood PMN were isolated from four healthy 12 to 15-week-old mice, as described in Duchez et al. [77], and pooled. The brain cortex (old cerebellum 3; OC3), were collected, after PBS washing, from exsanguinated 24-month-old C57BL/6 mice and flash-frozen in liquid nitrogen before storage at −80 °C. The brain cortex of 12 to 15-week-old C57BL/6 mice were used for the pull-down experiments.

#### 4.2.5. Cultured Mouse Cells

Neuronal N2a and NIH/3T3 fibroblast cell lines used in this study were obtained from ATCC (Manassas, VA, USA) and cultured according to ATCC’s recommendation.

#### 4.2.6. *Drosophila melanogaster*

Adult flies were purchased from the UCSD *Drosophila* Species Stock Center (San Diego) and snap-frozen in liquid nitrogen before storage at −80 °C.

#### 4.2.7. *Arabidopsis thaliana*

Samples of Arabidopsis total RNA were generously provided by Dr. Richard Bélanger (Université Laval).

#### 4.2.8. *Schizosaccharomyces pombe* and *Saccharomyces cerevisiae*

Total RNA samples from these two yeast strains were generously provided by Dr. Karl Ekwall (Karolinska Institutet).

### 4.3. Total RNA Isolation

Total RNA samples were prepared using TRIzol^®^ reagent or TRIzol LS^®^ reagent for liquid samples (Invitrogen Life Technologies, Carlsbad, CA, USA) following the manufacturer’s recommendations. Contaminating DNA was degraded by DNase I (M0303S, New England Biolabs, MA, USA) treatment following manufacturer’s instructions.

### 4.4. Small RNA Library and Sequencing

#### 4.4.1. Library Preparation

The quality control (QC) of our total RNA samples was assessed by gel electrophoresis and NanoDrop ND-1000 (Thermo Fisher Scientific, Waltham, MA, USA) analyses (Supplementary QC data). The small RNA-seq libraries, containing RNA species between 8 and 30 nt in length, were prepared as described previously [78]. Briefly, total RNA of each sample was used to prepare the small non-coding RNA sequencing library, which included the following steps: (1) 3′-adapter ligation, (2) 5′-adapter ligation, (3) cDNA synthesis, (4) PCR amplification, and (5) size selection of approximately 120 to 150 bp of PCR-amplified fragments (corresponding to approximately 8 to 30 nt of small RNA). The QC of the sequencing libraries was assessed by Agilent 2100 Bioanalyzer (Agilent Technologies, Waldbronn, Germany) (Supplementary QC data).

#### 4.4.2. sRNA-Seq

Samples were diluted to a final concentration of 8 pM, denatured as single-stranded DNA, and cluster generation was performed on the Illumina cBot using TruSeq Rapid SR cluster kit (GD-402-4001, Illumina, San Diego, CA, USA). Afterward, the clusters were sequenced for 51 cycles on Illumina HiSeq 2000 using TruSeq Rapid SBS Kits (FC-402-4002, Illumina, San Diego, CA, USA), as per the manufacturer’s instructions.

#### 4.4.3. Bioinformatics Analysis

Clean reads matching the quality standards were processed to remove the adaptor sequence, leading to sRNA trimmed reads. All reports displayed here were generated through the Arraystar Inc. (https://www.arraystar.com/ accessed on December 2015) standard analysis pipeline and refined using R (Free Software Foundation). Only the reads that were identical, both in length and sequence, were considered as a unique read. sRNA read counts were expressed as reads per million (RPM) sRNA alignments. Trimmed sRNA reads were mapped to the RefSeq database of the corresponding organism using the NCBI Basic Local Alignment Search Tool (BLAST) (https://blast.ncbi.nlm.nih.gov/Blast.cgi accessed on 2016).

### 4.5. Adapter-Ligated RT-qPCR Method

The splint and adaptor were annealed together, and RNA ligated retrotranscribed as described in our previous work Lambert et al. (manuscript submitted). qPCR was performed using miRCURY LNA SYBR^®^ Green PCR Kits (Qiagen Inc., Toronto, ON, Canada) in 96-well plates using the CFX96 Touch™ Real-Time PCR Detection System (Bio-Rad, Mississauga, ON, Canada) and specific Custom LNA Oligonucleotides [79,80] (Qiagen Inc., Toronto, ON, Canada). doRNA and C-doRNA. The small nucleolar RNA U6 was used as a reference gene for normalization, in compliance with the MIQE guidelines [81].

### 4.6. Fractionation Cytoplasm-Nucleus Analysis

Mouse neuronal N2a cells were washed with phosphate-buffered saline (PBS), lysed and fractionated into a cytoplasmic and a nuclear fraction, as described in the Supplementary Materials and Methods. One half of each fraction was used for Western blot analysis, and the other half for RT-qPCR quantification.

### 4.7. Pull-Down and Proteomics

Cleared lysates of mouse brain cortex were prepared, as described in Supplementary Materials and Methods. Three synthetic RNAs (doRNA, C-doRNA, and negative control; Integrated DNA Technologies, Inc., Coralville, IA, USA), biotinylated at their 3′ or 5′ end, were coupled to magnetic streptavidin beads (Dynabeads M-280 Streptavidin, Invitrogen Life Technologies, Carlsbad, CA, USA) and used to isolate proteins binding to each RNA. Proteins binding RNA were isolated using the previously prepared beads, washed and eluted at 95 °C in loading buffer. They were separated by 10% polyacrylamide gel electrophoresis and revealed with silver nitrate (Silver stain plus™ kit, Bio-Rad). Bands were excised and proteins were gel-extracted and identified by liquid chromatography-tandem mass spectrometry (LC/MS-MS) at the Proteomics Platform of the Eastern Quebec Genomics Center, Quebec City, QC, Canada, as described in Supplementary Materials and Methods.

### 4.8. Immunoprecipitation (IP)

Specific doRNA and/or C-doRNA-interacting proteins were immunoprecipitated using the following antibodies from Abcam (Cambridge, MA, USA): anti-hnRNP A0 (Rabbit polyclonal, ab157133), anti-hnRNP A1 (mouse monoclonal, ab5832), anti-hnRNP A2/B1 (Rabbit polyclonal, ab31645), anti-Ago2 (mouse monoclonal, ab3456), and normal immunoglobulin G from rabbit and mouse (IgG, Santa Cruz Biotechnology Inc., Paso Robles, CA, USA), as negative control. Anti-IgG-coated magnetic beads (Invitrogen Life Technologies, Carlsbad, CA, USA) were used to isolate the immune complexes.

### 4.9. Western Blot

We used the following primary antibodies from Abcam (Cambridge, MA, USA), unless cited otherwise, for Western blot detection: rabbit monoclonal anti-GAPDH (mouse monoclonal, ab9484), anti-PARP-1 (mouse monoclonal, C2-10; generously provided by Dr. Guy Poirier, Université Laval), anti-hnRNP A0 (rabbit polyclonal, ab157133), anti-hnRNP A1 (mouse monoclonal, ab5832), anti-hnRNP A2/B1 (rabbit polyclonal, ab31645) and anti-Ago2 (mouse monoclonal, ab3456). We used the following secondary antibodies from PerkinElmer (NEF812E001EA and NEF822E001EA Waltham, MA, USA): HRP-labeled anti-rabbit IgG and HRP-labeled anti-mouse IgG. Immunoreactive bands were visualized using a Clarity Max ECL detection kit (Bio-Rad, Mississauga, ON, Canada).

### 4.10. Transfection of Fluorescent RNA and Confocal Microscopy

doRNA, c-doRNA or negative control RNA, fluorescently labeled at their 3′ end with Cy3 (IDT, Coralville, IA), were transfected into cultured N2a cells using polyethylenimine (PEI) transfection agent (PEI, polyscience, Niles, IL, USA), as described in the Supplementary Materials and Methods. Then, 24 h later, cells were detached with trypsin (Thermo Fisher Scientific, Waltham, MA, USA), fixed for 10 min in formaldehyde 3% and applied to a microscopy slide with a cytospin (Thermo Fisher Scientific, Waltham, MA, USA). Cell nuclei were labeled with ProLong Diamond Antifade Mountant (SlowFade, S36942, Thermo Fisher Scientific, Waltham, MA, USA) with DAPI (Invitrogen, Burlington, ON, Canada), and cell membranes using green fluorescent PKH67 membrane-linker (Sigma Aldrich, Oakville, ON, Canada). Labeled cell nuclei, membranes and RNAs were visualized using a confocal microscope (Quorum spinning Disc Wave Fx, Quorum Technologies, Guelph, ON, Canada) with the 63× objective.

### 4.11. Dual-Luciferase Assay

Reporter gene activity assays were performed as previously described [78]. An annexin II receptor (AXIIR) 5′ UTR reporter construct was created by inserting a sequence of the AXIIR 5′ UTR downstream of the open reading frame encoding for the Renilla luciferase (Rluc) reporter gene of psiCHECK-2 vector (Promega, Madison, WI, USA). The constructs were verified by DNA sequencing. N2a cells were transfected 48 h prior to the measurement with 1 μg of DNA, while the doRNA, C-doRNA and negative RNA were transfected 24 h before. Rluc and Firefly luciferase (Fluc) activities were measured after washing the cells twice with 0.22-μm filtered sterile PBS and cell were lysed with Dual-Glo luciferase reagents (Promega, Madison, WI, USA), following the manufacturer’s instructions. Light emission was measured using a luminometer (Dynex Technologies, Chantilly, VA, USA). Renilla was used to normalize the values of the Firefly, since it did not contain an added DNA sequence.

### 4.12. Head Wound Assay (Scratch Assay)

LNCaP and RWPE-1 cells were seeded at 70% confluence and maintained in 6-well plates (SPL Life Sciences, Corp., Ltd., Korea) at 37 °C in a humidified atmosphere with 5% CO_2_. On the following day, cells were transfected with doRNA, C-doRNA or negative control RNA using PEI. After 24 h, cells were scratched with an SPL scar scratcher (SPL Life Sciences, Corp., Ltd., Korea). Images were captured at 40× *g* magnification using an Olympus CKX 41 microscope (Olympus, Corp., Tokyo, Japan), and analyzed using the eXcope 5.0.1 software to calculate the percentage of unrecovered scratched area at each time point (at 0 and 24 h).

### 4.13. Statistical Analysis

Statistical analyses were performed using Prism 7 (GraphPad Software, Inc., San diega, CA, USA). In vitro experiments were conducted in biological triplicates (minimum) with type alpha error set to 0.05 (5%). Statistical significance was determined by one or two-way ANOVA with Holm-Sidak’s post-hoc test for multiple comparisons or *t*-test.

## Figures and Tables

**Table 1:** Mapping of the doRNA and C-doRNA sequences to the human, mouse and fly transcriptomes using the nucleotide NCBI Basic Local Alignment Search Tool (BLAST) on NCBI database. The parameters used were the N blast, the standard database “nucleotide collection (nr/nt)” and the species “*Homo sapiens*,” or “*Mus musculus*,” or “*Drosophila melanogaster*.” The program used was BLASTN 2.12.0+. Results with 100% of identity and query coverage are shown in the table.

Description	% Identity	Accession No.
***Homo sapiens***		
*Homo sapiens* RNA, 5.8S ribosomal N3 (RNA5-8SN3), rRNA	100%	NR_146153.1
*Homo sapiens* RNA, 45S pre-ribosomal N2 (RNA45SN2), rRNA	100%	NR_146144.1
***Mus musculus***		
*Mus musculus* 5.8S rRNA	100%	K01367.1
*Mus musculus* 18S rRNA, 5.8S rRNA and 28S rRNA	100%	AH002077.2
***Drosophila melanogaster***		
*Drosophila melanogaster* pre-rRNA (pre-rRNA:CR45847), preRNA	100%	NR_133558.1
*Drosophila melanogaster* pre-rRNA (pre-rRNA:CR45846), preRNA	100%	NR_133554.1
*Drosophila melanogaster* pre-rRNA (pre-rRNA:CR45845), preRNA	100%	NR_133549.1
*Drosophila melanogaster* 5.8S rRNA (5.8SrRNA:CR45852)	100%	NR_133551.1
*Drosophila melanogaster* 5.8S and 2S rRNA	100%	U20145.1

Pre-rRNA, rRNA precursor; rRNA, ribosomal RNA.

## Data Availability

doRNA and C-doRNA sequences were deposited to the DNA Data Bank of Japan (entry ID for doRNA: 5f876b84a3c882000c844366; entry ID for C-doRNA: 5f876b84a3c882000c844366, both to be released soon). The small RNA-seq datasets generated and analyzed for the current study on doRNA and C-doRNA will be the subject of a separate report, until which time they are available from the corresponding author on a reasonable request. All raw small RNA-seq data generated in this study have been submitted to the NCBI Gene Expression Omnibus under accession number GSE179677. Instructions for Editors and Reviewers: Go to https://www.ncbi.nlm.nih.gov/geo/query/acc.cgi?acc=GSE179677 (accessed on 2 September 2021). Enter token mtyhesicpnunlyv into the box.

## Supplementary Materials

The following are available online at https://www.mdpi.com/article/10.3390/ijms22189757/s1.
