# Supplementary material for: Identification of Abundant and Functional dodecaRNAs (doRNAs) Derived from Ribosomal RNA
Source: Int J Mol Sci. 2021 Sep 9;22(18):9757. doi: 10.3390/ijms22189757 (PMC8467515; doi:10.3390/ijms22189757)
Supplement: Supplementary file 1 [file ijms-22-09757-s001.zip › ijms-1348259-supplementary/1 MS Lambert et al. doRNAs to IJMS Supplementary Materials and Methods.pdf]

SUPPLEMENTARY MATERIALS AND METHODS

Identification of new, abundant and functional dodecaRNAs (doRNAs) derived from ribosomal RNA

Marine Lambert[1,2,5], Abderrahim Benmoussa[1,2,5], Idrissa Diallo[1,2,5], Katheryn Ouellet-Boutin[1,2,5], Véronique Dorval[1†], Nathalie Majeau[1], Charles-Joly Beauparlant[1,3,5], Arnaud Droit[1,3,5], Alain Bergeron[1,4,5], Bernard Têtu[1,5], Yves Fradet[1,4,5], Frédéric Pouliot[1,4,5] , and Patrick Provost[1,2,5]*

[1] CHU de Québec-Université Laval Research Center/CHUL Pavilion, 2705 Blvd Laurier, Quebec City, QC, G1V 4G2, Canada; [2] Department of Microbiology, Infectious Diseases and Immunology, [3] Department of Molecular Medicine, [4] Department of Surgery, [5] Faculty of Medicine, Université Laval, Quebec City, QC, G1V 0A6, Canada

† Deceased

* To whom correspondence should be addressed: patrick.provost@crchudequebec.ulaval.ca

List of the medium used for cell culture

Cells	Medium
N2A	EMEM
NIH/3T3	DMEM
HEK293	DMEM
HUVEC	CCBM
RWPE-1	K-SFM
VCaP	DMEM
LaPC4	RPMI-1640
22Rv1	RPMI-1640
DU145	EMEM
PC-3	F-12K
LNCaP	RPMI-1640

Cytoplasmic-nuclear fractionation analysis

Neuronal N2a cells were washed with PBS, harvested in 300 µl hypotonic buffer (10 mM HEPES, 1.5 mM $MgCl_2$, 10 mM KCl, 1X Protease inhibitor, 40U Superase inhibitor, pH 7.9) , and incubated for 2 min on ice. At this step, 50 µl were saved to constitute the total control fraction. Thirty-two (32) µl of 10% NP-40 were added, and the sample was spun down (3,000 RPM, 850 g) for 7 min at 4°C. The volume of collected supernatant was measured and constituted the cytoplasmic fraction. The pellet was suspended in 300 µl of high salt buffer (30 mM HEPES, 0.4 mM EDTA, 1.5 mM $MgCl_2$, 800 mM NaCl, 1% NP-40, 1X Protease inhibitor, 40U Superase inhibitor, pH 7.9) followed by 30 min incubation on ice. After a final centrifugation (13,000 RPM, 16,000 g) during 30 min at 4°C, the supernatant, which constituted the nuclear fraction, was collected.

Pull-down and proteomics

Pull-down. Extracts from mouse brain were prepared in lysis buffer (25 mM Tris-HCl, 150 mM NaCl, 2 mM $MgCl_2$, 0.5% v/v Triton X-100, 5 mM DTT, 1X Protease inhibitor, 40U Superase inhibitor, pH 7.4) on ice using silicon beads and vortexing. To avoid non-specific interactions between the magnetic beads used for the pull-down and certain components of the samples, bovine serum albumin (BSA, Wisent, QC, Canada) was added to the mix to saturate those non-specific sites and limit background signal. Cleared extracts were brought into contact with synthetic biotinylated RNA (doRNA, C-doARN or negative control RNA). Two hours later, magnetic beads coupled to streptavidin were added for one additional hour and incubated under continuous rotation at 4°C. After several washes in lysis buffer (25 mM Tris-HCl, 150 mM NaCl, 2 mM $MgCl_2$, 0.5% v/v Triton X-100, 5 mM DTT, 1X Protease inhibitor, 40U Superase inhibitor, pH 7.4), then in low-triton buffer (25 mM Tris-HCl, 150 mM NaCl, 2 mM $MgCl_2$, 0.05% v/v Triton X-100, 5 mM DTT, 1X Protease inhibitor, 40U Superase inhibitor, pH 7.4) proteins that remained attached to the beads were eluted.

Protein identification by mass spectrometry (MS). Proteins were extracted from the gel and resuspended in a 0.5% Deoxycholate (DOC) buffer (0.5% DOC, 50 mM ammonium bicarbonate, 50 mM DTT, 1X protease inhibitor, 1 µM pepstatin). MS was performed by the Proteomics Platform of the Eastern Quebec Genomics Center, Quebec, Canada (http://www.crchudequebec.ulaval.ca/en/services/proteomics/about-us/). Typically, proteins were digested with trypsin before injecting the digested peptides into a capillary HPLC (high performance liquid chromatography) system linked to a high resolution and high accuracy mass spectrometer (TripleTOF 5600+ or an Orbitrap Fusion). Peptides were separated by online reverse-phase (RP) nanoscale capillary liquid chromatography (nanoLC) and analyzed by electrospray MS (ES-MS). The results were interpreted to provide statistically validated matches between observed spectra and identified peptides and a list of proteins depending on the species. Protein identification was confirmed if it could be established at >99% probability and contained at least two identified peptides.

Western blot analysis

Western blot analysis. Proteins were denatured in sample buffer (62.5 mM Tris-HCl, 2% w/v SDS, 10% v/v glycerol, 50 mM DTT and 0.01% Bromophenol Blue, pH 6.8) separated by 12% polyacrylamide gel electrophoresis and electrotransferred onto a polyvinylidene difluoride (PVDF) membrane.

Transfection of florescent RNA and confocal microscopy

Transfection. Briefly, polyethylenimine (PEI, Polyscience, PA) powder was diluted in sterilized water at a 1 mg/ml concentration, filtered, and aliquoted. One µl of PEI and 100 nmole of RNA were separately spiked in 100 µl of EMEM medium without serum and antibiotic, left for 5 min for diffusion and then the two solutions were mixed. Twenty-five min later, the RNA•PEI mixtures were added to cells grown to 60% confluence in 6-well plates (1). RNA labeled with Cy3 at their 3' end were ordered from IDT (Coralville, IA).

Supplementary Reference

1. Longo,P.A., Kavran,J.M., Kim,M.-S. and Leahy,D.J. (2013) Transient mammalian cell transfection with polyethylenimine (PEI). *Methods Enzymol.*, **529**, 227–240.
