# Supplementary material for: Identification of Abundant and Functional dodecaRNAs (doRNAs) Derived from Ribosomal RNA
Source: Int J Mol Sci. 2021 Sep 9;22(18):9757. doi: 10.3390/ijms22189757 (PMC8467515; doi:10.3390/ijms22189757)
Supplement: Supplementary file 1 [file ijms-22-09757-s001.zip › ijms-1348259-supplementary/3 MS Lambert et al. doRNAs to IJMS Supplementary Figures.pdf]

SUPPLEMENTARY FIGURES

Identification of Abundant and Functional dodecaRNAs (doRNAs) Derived from Ribosomal RNA

Marine Lambert [1,2,3] Abderrahim Benmoussa [1,2,3], Idrissa Diallo [1,2,3], Katheryn Ouellet-Boutin [1,2,3], Véronique Dorval [1,†], Nathalie Majeau [1], Charles Joly Beauparlant [1,3,4], Arnaud Droit [1,3,4,], Alain Bergeron [1,3,5], Bernard Têtu [1,3], Yves Fradet [1,3,5], Frédéric Pouliot [1,3,5] and Patrick Provost [1,2,3,*]

1. CHU de Québec-Université Laval Research Center/CHUL Pavilion, 2705 boulevard Laurier, Quebec City, QC G1V 4G2, Canada; marine.lambert@mail.mcgill.ca (M.L.); abderrahim.benmoussa@umontreal.ca (A.B.); Idrissa.Diallo@crchudequebec.ulaval.ca (I.D.); katheryn.ouellet-boutin@crchudequebec.ulaval.ca (K.O.-B.); nathalie.majeau@crchudequebec.ulaval.ca (N.M.); Charles.Joly-Beauparlant@crchudequebec.ulaval.ca (C.J.B.); arnaud.droit@crchudequebec.ulaval.ca (A.D.); Alain.Bergeron@crchudequebec.ulaval.ca (A.B.); bernard.tetu@fmed.ulaval.ca (B.T.); Yves.Fradet@crchudequebec.ulaval.ca (Y.F.); Frederic.pouliot@crchudequebec.ulaval.ca (F.P.)
2. Department of Microbiology, Infectious Diseases and Immunology
3. Faculty of Medicine, Université Laval, Quebec City, QC G1V 0A6, Canada
4. Department of Molecular Medicine
5. Department of Surgery

* Correspondence: patrick.provost@crchudequebec.ulaval.ca

† Deceased.

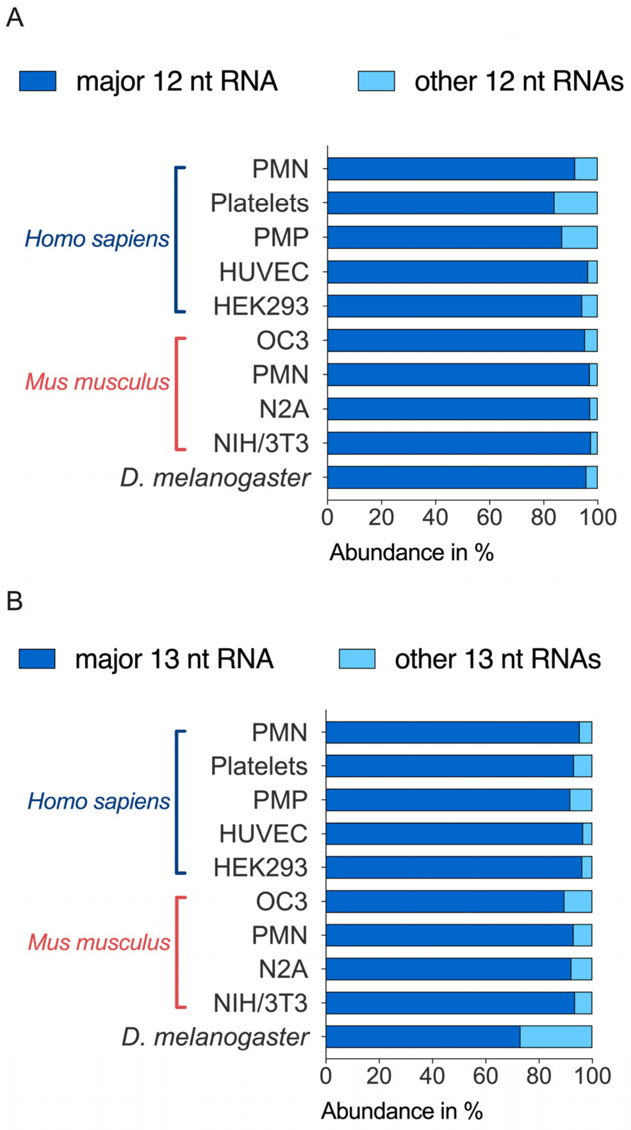

Supplementary Figure S1. One sequence represents most of the 12-nt RNA reads and one for the 13-nt RNA reads in our small RNA-seq data. (**A**) More than 80% of the 12-nt RNA reads come from one unique RNA in cells from *H. sapiens*, *M. musculus* and *D. melanogaster*. (**B**) More than 70% of the 13-nt RNA reads come from one unique RNA in cells from *H. sapiens*, *M. musculus* and *D. melanogaster*. PMN, polymorphonuclear leukocytes; PMP, platelet-derived microparticles; HUVEC, human umbilical vein endothelial cells; HEK293, human embryonic kidney 293 cells; OC3, Old Cerebellum 3; N2a, mouse neuroblastoma cells; NIH/3T3, mouse embryonic fibroblast cells.

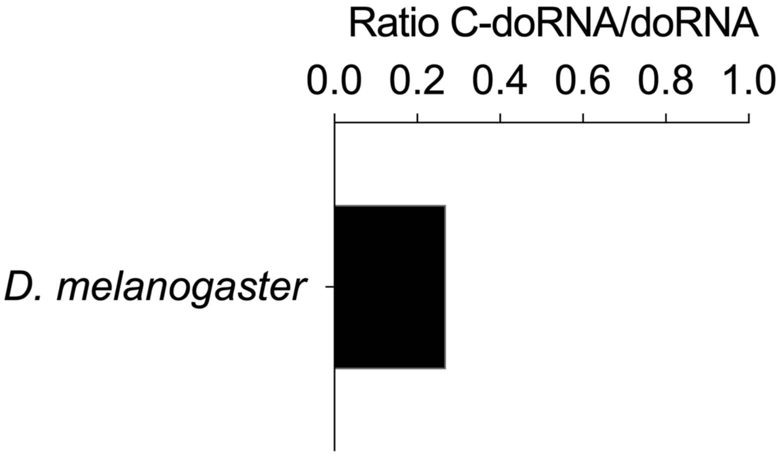

Supplementary Figure S2. Calculated C-doRNA/doRNA ratio in *Drosophila melanogaster* derived from small RNA-Seq data. The calculated C-doRNA/doRNA ratio was at 0.27.

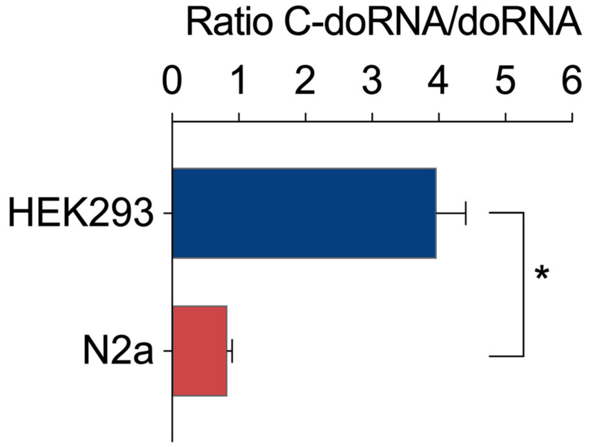

Supplementary Figure S3. The C-doRNA/doRNA ratio was significatively higher in HEK293 cells, compared to N2a cells. The C-doRNA/doRNA ratio was calculated from splinted ligation RT-qPCR analysis of total RNA (mean ± SEM; n=3 independent experiments). * $p < 0.05$, one-way ANOVA with Holm Sidak's post-hoc test.

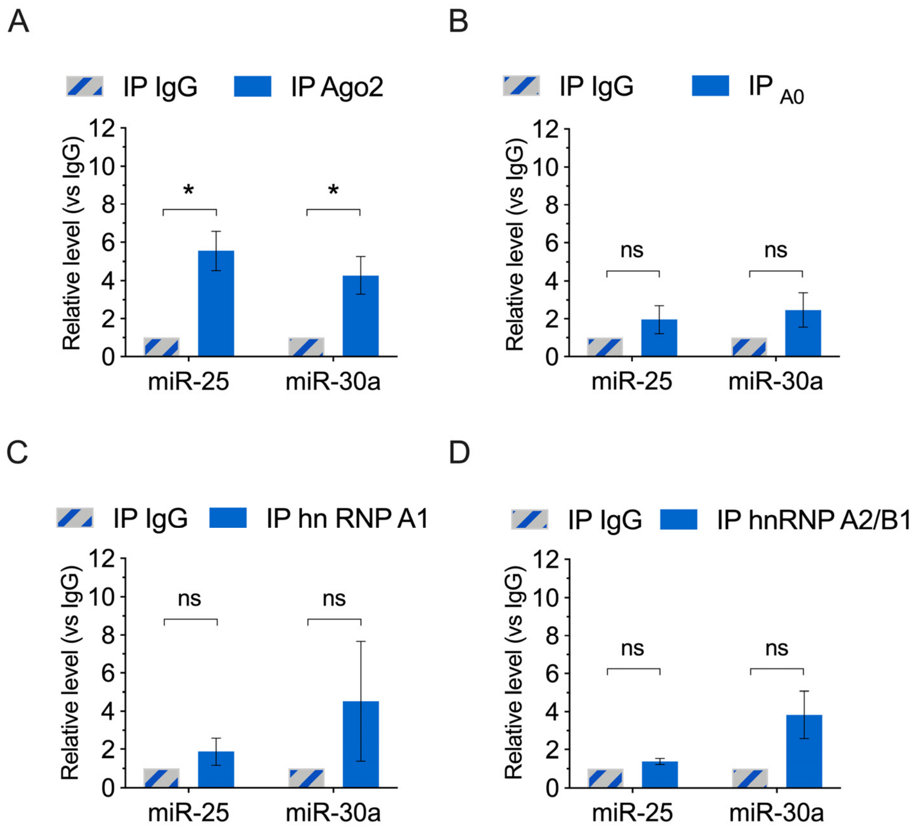

Supplementary Figure S4. MicroRNAs miR-25 and miR-30a are associated with Ago2 proteins, but not to hnRNP A0, A1 and A2B1 proteins. hnRNP A0, hnRNP A1, hnRNP A2B1 or Ago2 proteins were immunoprecipitated from mouse brain extracts, followed by total RNA extraction and RT-qPCR detection of miR-25 and miR-30a. The copy number of miR-25 and miR-30a was calculated using a standard curve established using synthetic microRNAs. The results are shown as fold change vs the IgG immunoprecipitation (mean ± SEM; n = 3 independent experiments). * p value < 0.05. Two-way ANOVA, Holm and Sidak's multiple comparisons post-hoc test. A1, hnRNP A1; A0, hnRNP A0; A2/B1, hnRNP A2B1; Ago, Argonaute.

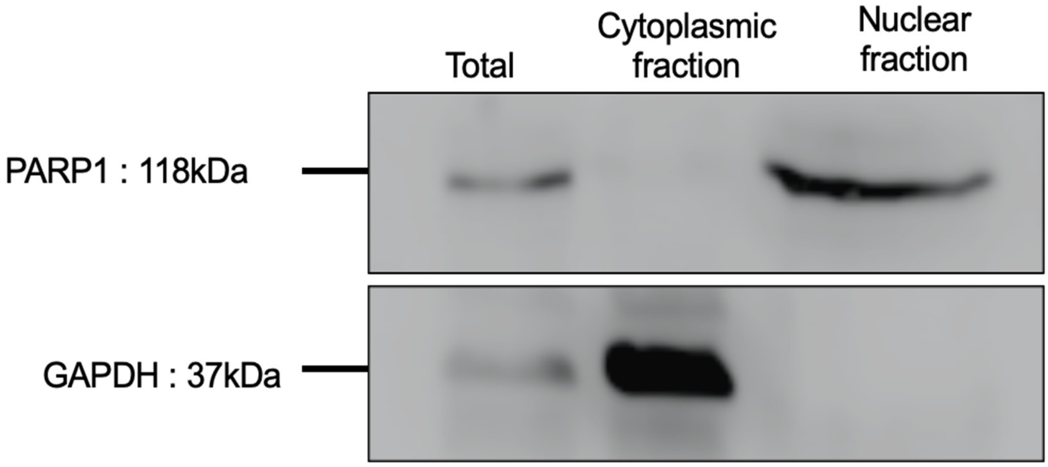

Supplementary Figure S5. Subcellular fractionation was verified by Western blot. Cultured N2a cell fractionation was verified by Western blotting using GAPDH as a cytoplasmic protein marker and PARP1 as the nuclear protein marker. Blot representative of 3 independent experiments.

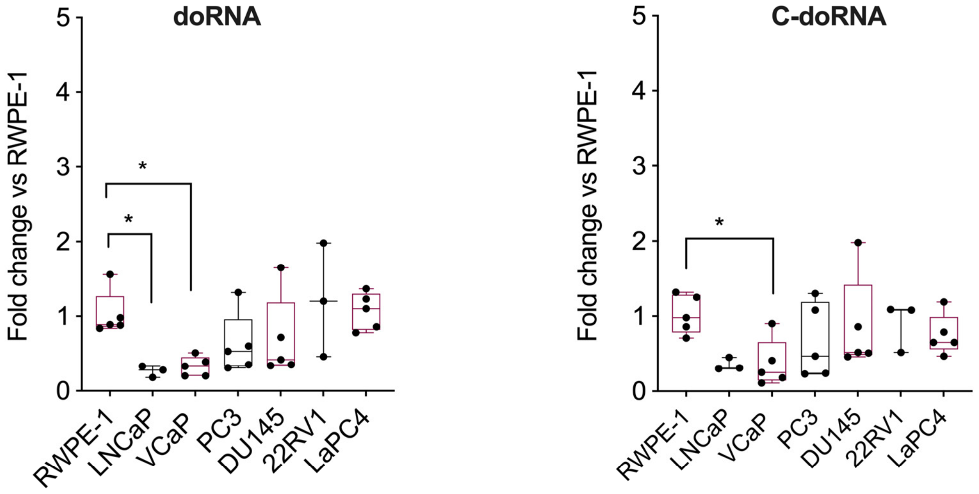

Supplementary Figure S6. doRNA and C-doRNA levels tend to be reduced in LNCaP and VCaP pancreatic cancer cell lines. doRNA and C-doRNA levels in one normal and six cancerous prostate cell lines were quantitated by splinted 5' ligation RT-qPCR. The data were expressed as fold change using the level in the normal prostate cell line RWPE-1 as a reference (mean ± SEM; n = 5 independent experiments for RWPE-1, VCaP, PC3, DU145, and LaPC4 cells; n = 3 independent experiments for LNCaP and 22RV1). * $p < 0.05$. One-way ANOVA with Holm Sidak's post-hoc test.

Human 5.8S rRNA (U13369)

Show All

```
  1  CGACUCUUAG CGGUmGGAUCA CUCGGCUCGU GCGUCGAUGA AGAACGCAGC
                  HBII-239

 51  UAGCΨGCGAG AAUUAAUGΨG AAUUGmCAGGA CACAUUGAUC AUCGACACUU
        U72             U69  U96a  U96b

100  CGAACGCACU UGCGGCCCCG GGUUCCUCCC GGGGCUACGC CUGUCUGAGC

151  GUCGCUU
```

Supplementary Figure S7. Chemical modifications of nucleotides in the human 5.8S rRNA sequence. Uracil 14 (U14) methylation may be induced by snoRNA HBII-239 (MBII-239 in mice). Information from the “3D RIBOSOMAL MODIFICATION MAPS DATABASE” (1) (Piekna-Przybylska *et al.,* 2008).

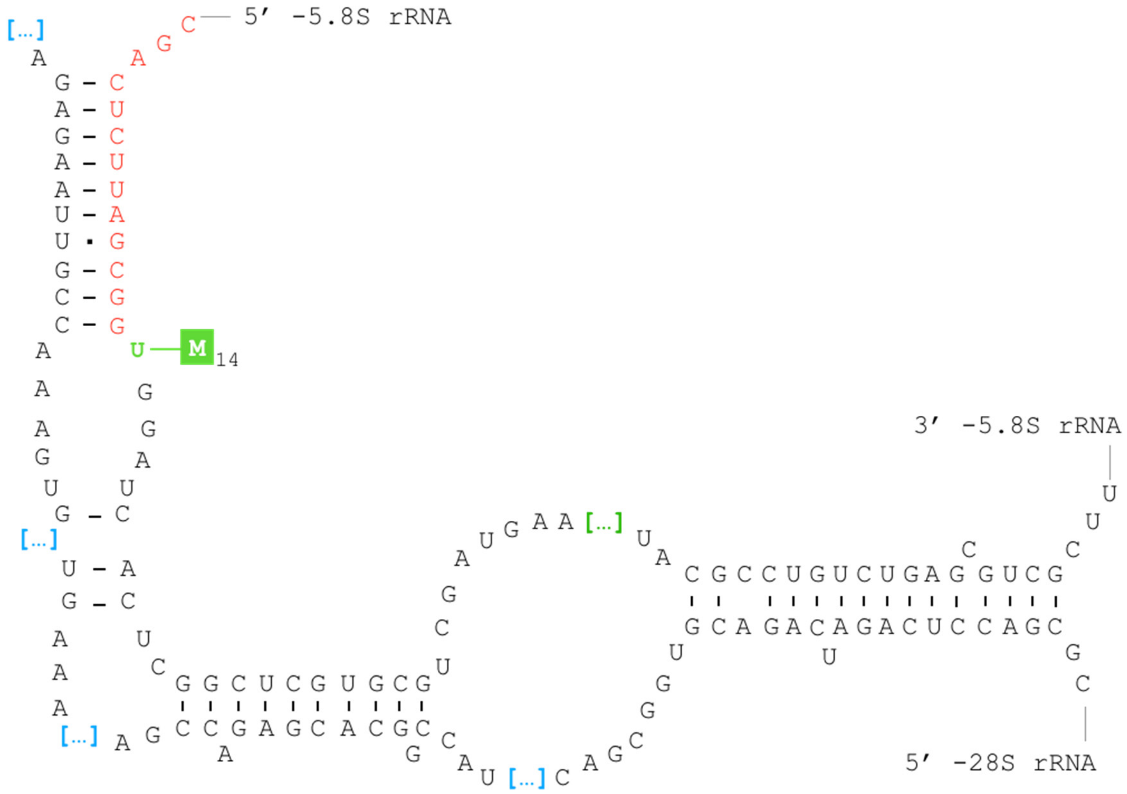

Supplementary Figure S8. Schematic representation of 5.8S and 28S rRNA interactions and binding sequences in higher eukaryotes. 5.8S rRNA U14 methylation is depicted in green. The C-doRNA sequence is colored in red. Information from the "3D RIBOSOMAL MODIFICATION MAPS DATABASE" (1) (Piekna-Przybylska *et al.*, 2008).

Supplementary Reference

1. Piekna-Przybylska,D., Decatur,W.A. and Fournier,M.J. (2008) The 3D rRNA modification maps database: with interactive tools for ribosome analysis. *Nucleic Acids Res.*, **36**, D178-83.
